# Tumor Number and Model for End‐Stage Liver Disease Score as Selection Criteria for Resection or Embolization of Intermediate‐Stage Hepatocellular Carcinoma

**DOI:** 10.1002/cnr2.70697

**Published:** 2026-09-20

**Authors:** Yi‐Hao Yen, Chee‐Chien Yong, Yueh‐Wei Liu, Wei‐Feng Li, Chih‐Chi Wang, Chih‐Yun Lin

**Affiliations:** ^1^ Division of Hepatogastroenterology, Department of Internal Medicine, Kaohsiung Chang Gung Memorial Hospital Chang Gung University College of Medicine Kaohsiung Taiwan; ^2^ Department of Surgery, Liver Transplantation Center Kaohsiung Chang Gung Memorial Hospital Kaohsiung Taiwan; ^3^ Biostatistics Center of Kaohsiung Chang Gung Memorial Hospital Kaohsiung Taiwan

**Keywords:** Barcelona clinic liver cancer stage, hepatocellular carcinoma, liver resection, overall survival, transarterial chemoembolization

## Abstract

**Background:**

Tumor and liver‐related factors are well‐known prognostic factors for patients with hepatocellular carcinoma (HCC). The Japanese Society of Hepatology (JSH) guidelines recommend considering liver resection (LR) for patients with multiple tumors and a tumor number ≤ 3. A previous Italian study showed that a model for end‐stage liver disease (MELD) score of > 9 indicates inadequate liver function reserve in patients with HCC undergoing LR.

**Aims:**

We used these two parameters to predict the overall survival (OS) of patients with Barcelona clinic liver cancer (BCLC) Stage B HCC who underwent LR or transcatheter arterial chemoembolization (TACE).

**Methods and Results:**

We consecutively enrolled patients with BCLC stage B HCC and Child–Pugh Class A liver disease underwent LR or TACE. Of these patients, 163 underwent LR and 270 underwent TACE. We used two parameters, that is, ≤ 3 nodules and a MELD score of ≤ 9, to subclassify BCLC stage B HCC patients. BCLC B1 had to occur concomitantly with two parameters, whereas for BCLC B2, meeting only one criterion was sufficient. The five‐year OS of BCLC B1 patients undergoing LR was 60% and those undergoing TACE was 37% (*p* = 0.007). The 5‐year OS of BCLC B2 patients undergoing LR was 23% and those undergoing TACE was 18% (*p* = 0.223).

**Conclusion:**

We recommend considering LR for BCLC B1 patients, whereas TACE could be considered for BCLC B2 patients.

## 1 Introduction

Hepatocellular carcinoma (HCC) is a leading cause of cancer‐related death in Taiwan as well as worldwide [1]. The Barcelona clinic liver cancer (BCLC) guidelines recommend transarterial chemoembolization (TACE) for patients with BCLC stage B HCC [2]. Several treatment modalities are available for HCC, including radioembolization, multipolar radiofrequency ablation (RFA), microwave ablation, and systemic therapies, which can be applied to treat BCLC stage B HCC. However, liver resection (LR) still plays an important role because of its advantage of removal of large tumors, a benefit that might not be offered by local regional or systemic therapies [2, 3].

Tumor and liver‐related factors are well‐known prognostic factors for patients with HCC [2, 3]. The Japanese Society of Hepatology (JSH) guidelines recommend considering LR for patients with multiple tumors and a tumor number ≤ 3, whereas non‐surgical treatments for > 3 tumors [4]. A previous Italian study showed that a model for end‐stage liver disease (MELD) score of > 9 indicates inadequate liver function reserve in patients with HCC undergoing LR [5].

Therefore, in this retrospective study, we aimed to use these two parameters to determine the ideal candidates for LR among patients with BCLC stage B HCC. We enrolled a cohort of patients with BCLC B HCC and Child–Pugh Class A liver disease undergoing LR or TACE. We used two parameters, that is, ≤ 3 nodules and a MELD score of ≤ 9, to subclassify BCLC stage B HCC patients. BCLC B1 had to occur concomitantly with two parameters, whereas for BCLC B2, meeting only one criterion was sufficient. We compared overall survival (OS) between patients undergoing LR and those receiving TACE in the two groups.

## 2 Methods

### 2.1 Ethical Approval

The protocol for the research project has been approved by Kaohsiung Chang Gung Memorial Hospital (Reference Number: 202201189B0) within which the work was undertaken and that it conforms to the provisions of the Declaration of Helsinki. The requirement of informed consent was waived by the Institutional Review Board of Kaohsiung Chang Gung Memorial Hospital (Reference Number: 202201189B0).

### 2.2 Treatment Modality Decisions for Patients With BCLC B HCC and Child–Pugh Class A Liver Disease

Each patient was discussed in a multidisciplinary tumor board. In general, liver transplantation is an option for patients with a tumor burden within the University of California San Francisco criteria [6] and a low alpha‐fetoprotein (AFP) level (no definite cutoff value) and clinically significant portal hypertension (CSPH), that is, a platelet count < 10^9^/L with splenomegaly, presence of varices, or portosystemic collateral vessels [7]. Lobectomy is an option for patients with tumors located in one lobe; in contrast, for patients with bilobar tumors, LR for the main tumor(s) plus intraoperative RFA or preoperative TACE for the remaining small nodules is an option. TACE is an option for patients without advanced kidney dysfunction. RFA with single needle is applied for multiple tumors < 3.0 cm, and multipolar RFA for multiple tumors with one of them 3.0–5.0 cm. In addition, TACE followed by RFA is an option for patients with residual tumors that could be completely ablated. Although the subgroup of patients benefiting from selective internal radiation therapy (SIRT) with yttrium‐90 needs to be defined [3], SIRT is used in preference to TACE at our institution for patients with larger tumors. External beam radiation, systemic chemotherapy, or target therapy, that is, sorafenib or lenvatinib, is an option for infiltrative hypovascular tumors or high tumor burden not eligible for TACE (as mentioned in the European Association for the Study of the Liver [EASL] guidelines, a tumor burden > 50% of total liver volume increases the risk of hepatic decompensation after TACE [8, 9]). Target therapies and SIRT are not reimbursed by Taiwan's National Health Insurance for patients with BCLC B HCC.

### 2.3 Enrollment of Patients

**FIGURE 1:**
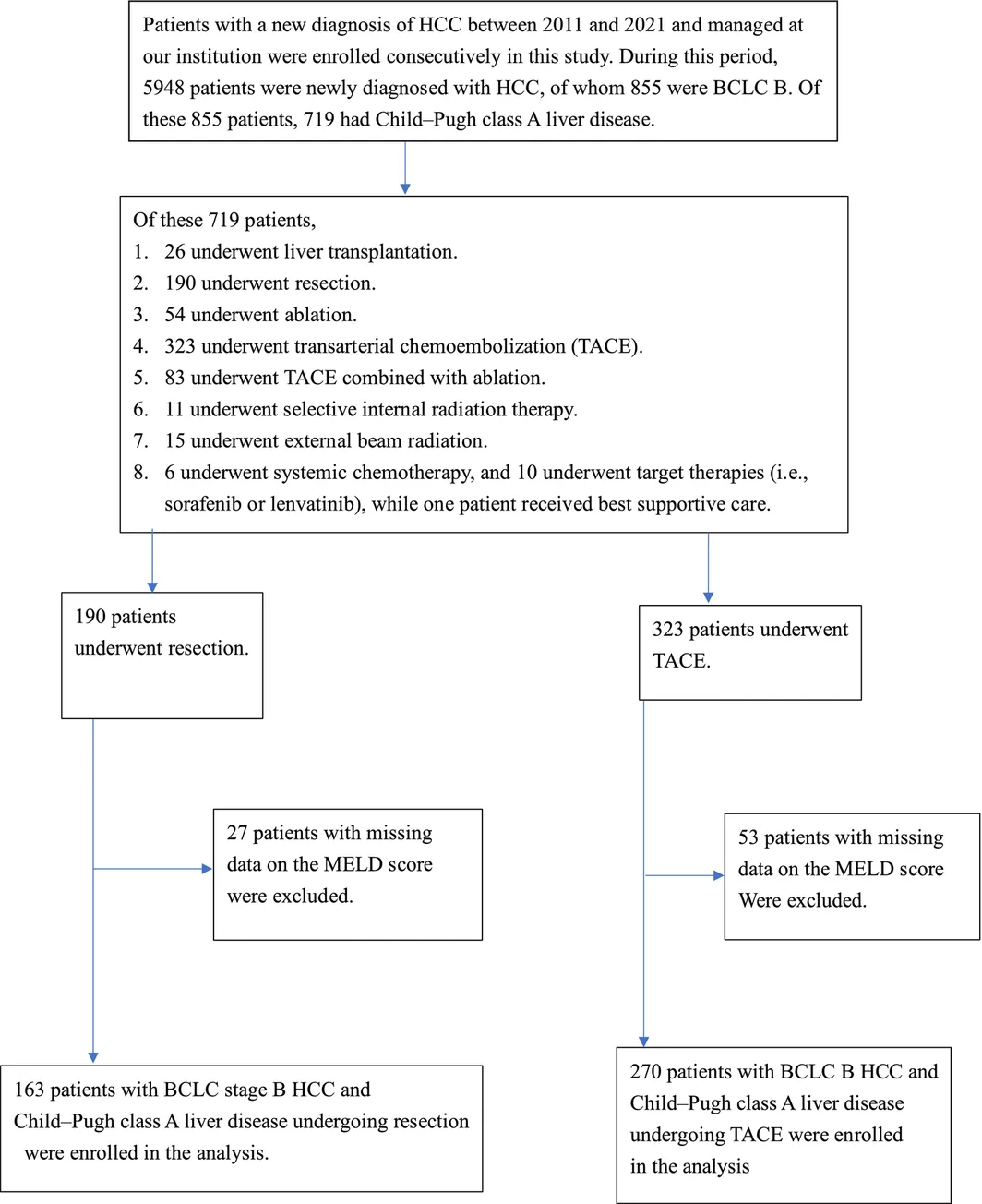
The flowchart of patient enrollment.

Patients with a new diagnosis of HCC between 2011 and 2021 and managed at our institution were enrolled consecutively in this study. Data were retrieved from the HCC registry kept by our institution. The flowchart of patient enrollment is shown in Figure 1.

### 2.4 Variables of Interest

Age, sex, AFP level, hepatitis B virus surface antigen (HBsAg) and anti‐hepatitis C virus (HCV) antibody test results, total bilirubin, creatinine, international normalized ratio (INR), Child–Pugh classification [10], and MELD score were variables of interest [11]. BCLC stage B patients are those with Child–Pugh A or B liver disease with multifocal HCC beyond Milan criteria [12] who do not have cancer‐related symptoms and do not show macrovascular invasion or extrahepatic spread [13]. Tumor size and number were determined from contrast enhanced computed tomography or magnetic resonance imaging results.

### 2.5 Cutoffs for Radiological Tumor Size, Number, Serum AFP Level, and MELD Score

A tumor size cutoff of 5 cm was used per the AJCC staging system [13, 14]. The JSH guidelines recommend LR for patients with 2–3 tumors and non‐surgical treatment for those with > 3 tumors [4]. Therefore, we used a tumor number cutoff of > 3. An AFP cutoff of ≥ 400 ng/mL was used per the Cancer of the Liver Italian Program (CLIP) staging [15]. A MELD score cutoff of > 9 was used according to a previous Italian study, which enrolled 543 patients with HCC undergoing LR. Multivariate analysis showed that a MELD score of > 9 (odds ratio = 2.26, 95% CI = 1.10–4.58, *p* = 0.02) was independently associated with postoperative liver decompensation [5]. Therefore, we believed a MELD score of > 9 may indicate inadequate liver function reserve for patients with HCC undergoing LR.

### 2.6 Definition of Ideal Candidates for Liver Resection Among Patients With BCLC B HCC

According to the JSH guidelines recommendation: considering LR for patients with multiple tumors and a tumor number ≤ 3, whereas non‐surgical treatments for > 3 tumors [4]. Based on a previous Italian study [5], we defined a MELD score of ≤ 9 as well‐preserved liver function. Thus, we used two parameters, that is, ≤ 3 nodules and a MELD score of ≤ 9, to subclassify BCLC stage B HCC patients. In BCLC B1 patients, HCC had to occur concomitantly with the two parameters (defined as the ideal candidates for LR), whereas for BCLC B2 patients, meeting only one criterion was sufficient (defined as nonideal candidates for LR).

### 2.7 Outcome Measurement

The primary outcome measurement was OS, which was calculated as the time from the date of LR or TACE to the date of the last follow‐up or death.

### 2.8 Statistical Analyses

Patient characteristics are presented as number (%). Categorical variables were analyzed using the chi‐square test. The Kaplan–Meier estimator and log‐rank test were used to compare OS between groups. All *p* values were two‐tailed, and a *p* value of < 0.05 was considered statistically significant. All statistical analyses were performed using the computing environment IBM SPSS Statistics, version 25.

## 3 Results

### 3.1 Characteristics of Patients With BCLC B1 and B2 Hepatocellular Carcinoma According to Treatment Modality

Of the 148 patients with BCLC B1 HCC, 84 underwent LR and 64 underwent TACE. The proportion of patients showing anti‐HCV positivity was lower in the LR subgroup than in the TACE subgroup (*p* = 0.046), whereas age, sex, the diameter of the largest nodule, AFP level, and HBsAg positivity did not differ significantly (Table 1). Of the 285 patients with BCLC B2 HCC, 79 underwent LR and 206 underwent TACE. The two patient subgroups did not differ in age, sex, the diameter of the largest nodule, AFP level, HBsAg and anti‐HCV positivity, and MELD score > 9. However, the proportion of patients with > 3 tumors was higher in the TACE subgroup than in the LR subgroup (*p* < 0.001) (Table 2).

**TABLE 1:** Characteristics of patients with Barcelona clinic liver cancer B1.

	LR, *n* = 84	TACE, *n* = 64	*p*
Age > 65 years	27 (32.1%)	30 (46.9%)	0.068
Men	66 (78.6%)	51 (79.7%)	0.869
The diameter of the largest nodule > 5.0 cm	45 (53.6%)	24 (37.5%)	0.052
HBsAg positive	47 (56.0%)	32 (50.0%)	0.472
Anti‐HCV positive	20 (23.8%)	25 (39.1%)	0.046
AFP ≧ 400 ng/mL	25 (29.8%)	12 (18.8%)	0.125

Abbreviations: AFP, alpha‐protein; HBsAg, hepatitis B surface antigen; HCV, hepatitis C virus; LR, liver resection; TACE, transarterial chemoembolization.

**TABLE 2:** Characteristics of patients with Barcelona clinic liver cancer B2.

	LR, *n* = 79	TACE, *n* = 206	*p*
Age > 65 years	27 (34.2%)	75 (36.4%)	0.725
Men	66 (83.5%)	162 (78.6%)	0.354
The diameter of the largest nodule > 5.0 cm	49 (62.0%)	104 (50.7%)	0.087
HBsAg positive	51 (64.6%)	108 (52.4%)	0.065
Anti‐HCV positive	19 (24.1%)	62 (30.1%)	0.311
AFP ≧ 400 ng/mL	29 (36.7%)	66 (32.0%)	0.454
MELD score > 9	45 (57.0%)	102 (49.5%)	0.260
Tumor number > 3	44 (55.7%)	163 (79.1%)	< 0.001

Abbreviations: AFP, alpha‐protein; HBsAg, hepatitis B surface antigen; HCV, hepatitis C virus; LR, liver resection; MELD, model for end‐stage liver disease; TACE, transarterial chemoembolization.

### 3.2 Five‐Year Overall Survival of Patients According to BCLC B Substage and Treatment Modality

**FIGURE 2:**
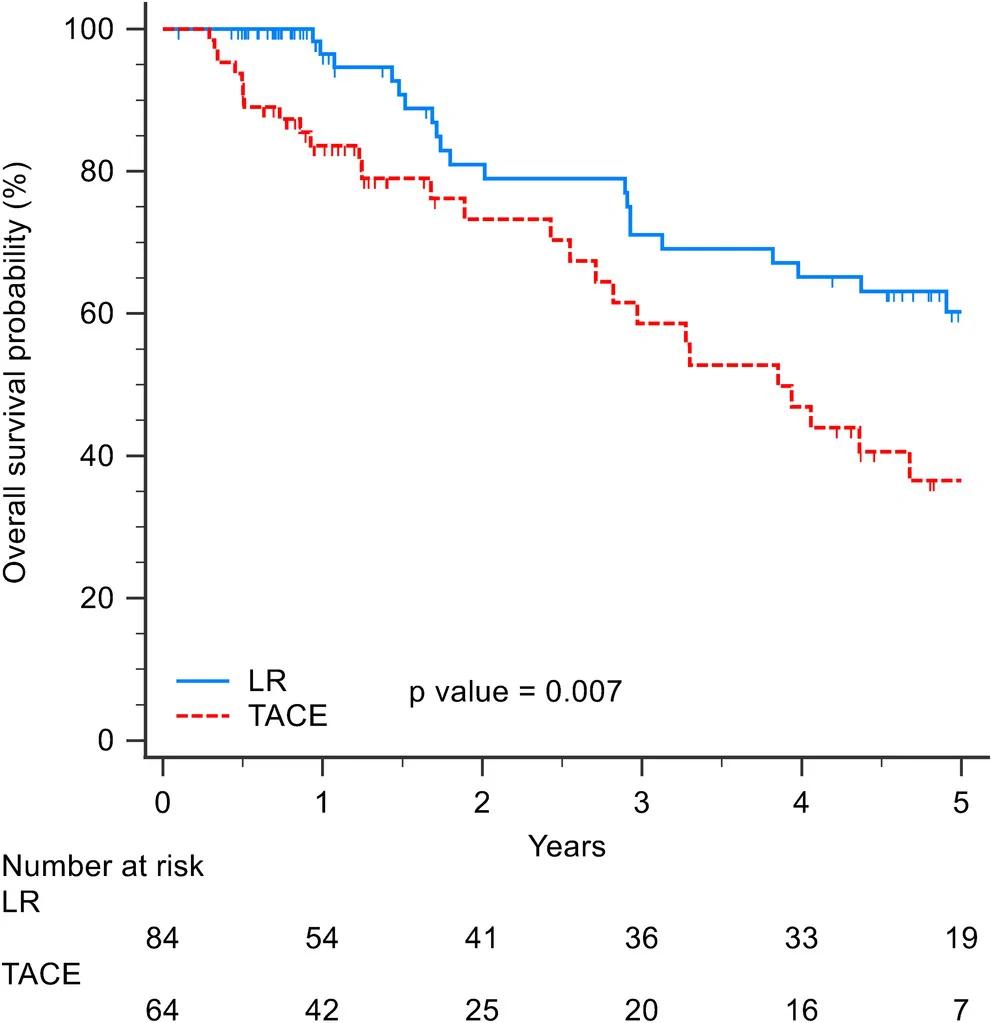
Overall survival of patients with Barcelona clinic liver cancer Stage B1 hepatocellular carcinoma, that is, ≤ 3 nodules and a model for end‐stage liver disease score of ≤ 9, according to treatment modality. The 5‐year overall survival of patients undergoing liver resection was 60% and that of patients undergoing transarterial chemoembolization was 37% (*p* = 0.007).

**FIGURE 3:**
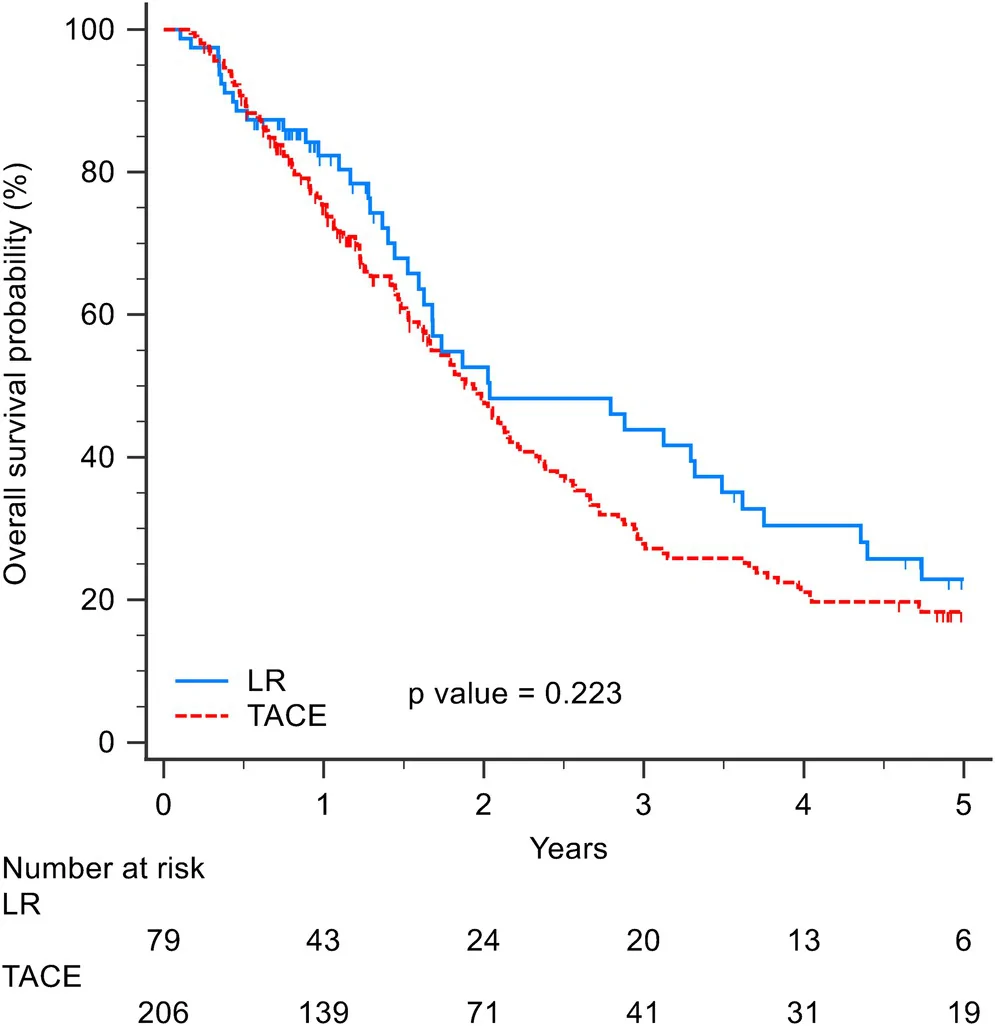
Overall survival of patients with Barcelona clinic liver cancer Stage B2 hepatocellular carcinoma, that is, > 3 nodules or a model for end‐stage liver disease score of > 9, according to treatment modality. The 5‐year overall survival of patients undergoing liver resection was 23% and that of patients undergoing transarterial chemoembolization was 18% (*p* = 0.223).

In the BCLC B1 group, the five‐year OS of patients undergoing LR was 60% and that of patients receiving TACE was 37% during a median follow‐up of 1.4 years (IQR = 0.8–3.4 years). In this group, the median OS was not reached by patients undergoing LR and it was 3.9 (IQR = 2.8–4.9) years among patients receiving TACE (*p* = 0.007) (Figure 2). No patients died within 90 days of treatment in this group. In the BCLC B2 group, the five‐year OS of patients undergoing LR was 23% and that of patients undergoing TACE was 18%. In this group, the median OS was 2.0 (IQR = 0.8–3.3) years and 1.9 (IQR = 1.6–2.2) years in the LR and TACE subgroups, respectively (*p* = 0.223) (Figure 3). In addition, in the BCLC B2 group, two (2.5%) patients undergoing LR and four (1.9%) patients receiving TACE died within 90 days of treatment (*p* = 0.671).

## 4 Discussion

In the present study, we used two parameters, that is, ≤ 3 nodules and a MELD score of ≤ 9, to subclassify BCLC stage B HCC patients. BCLC B1 had to occur concomitantly with two parameters, whereas for BCLC B2, meeting only one criterion was sufficient. We found that patients with BCLC B1 are ideal candidates for LR, whereas those with BCLC B2 are not. This simple model would be useful for clinical practice.

The number of BCLC B patients who underwent resection is noteworthy in the present study. Typically, BCLC B patients are not considered candidates for surgical resection. The advantage of LR is the removal of large tumors, a benefit that might not be offered by RFA or TACE [2, 3]. The diameter of the largest nodule was > 5.0 cm in 94 (57.6%) of the 163 patients who underwent surgical resection in the present study.

We enrolled patients with Child–Pugh Class A liver disease in the present study. Traditionally, Child–Pugh Class A liver disease is subclassified as Child–Pugh score 5 or 6, with or without CSPH and albumin–bilirubin Grade 1 or 2/3 [2]. Our HCC registry data lacked the above‐mentioned data. Therefore, we used a MELD score of > 9 or ≤ 9 to subclassify Child–Pugh Class A liver disease.

Traditionally, the MELD score is used to evaluate the severity of liver function reserve in patients with liver decompensation [16]. However, numerous studies have shown its utility in patients with HCC undergoing LR [17, 18, 19, 20, 21], further supporting the feasibility of the application of the MELD score for patients undergoing LR for HCC.

Increased tumor number is a well‐known factor of poor prognosis of patients with HCC [2, 3]. Wang et al. reported that a higher tumor number is associated with microvascular invasion in patients with BCLC B HCC undergoing LR, which could explain this phenomenon [22].

A Japanese study enrolling 3246 patients (LR: *n* = 1944; TACE: *n* = 1302) with ≤ 3 tumor nodules and Child–Pugh Class A liver disease found that after propensity score matching (PSM), the OS of the LR group was 60% at 5 years, which was higher than that of the TACE group (41.6%, *p* < 0.001) [23]. This nationwide survey from Japan further supports the results of the present study.

Bolondi et al. [24] and Kudo et al. [25] proposed subclassification of BCLC B HCC due to the heterogeneity of the disease at this stage. Both teams subclassified BCLC B according to the Child–Pugh score (i.e., 5–7 vs. 8–9) and tumor burden (i.e., within or out of up‐to‐seven, seven representing the sum of tumor number and the largest tumor size [cm]) [24, 25].

In a Spanish study enrolling 80 patients with BCLC B HCC, 44 patients underwent TACE and 36 underwent LR. The authors classified the patients according to Bolondi's criteria. The five‐year recurrence rate was 25% for LR and 60% for TACE in the B1 group (*p* = 0.018) defined as having a Child–Pugh score of 5–7 and within up‐to‐seven, whereas the survival outcome was not different between other BCLC subgroups [26]. A Japanese study classified patients with BCLC B HCC (*n* = 378) according to the Kinki criteria (proposed by Kudo et al. [25]). B1 was defined as having a Child–Pugh score of 5–7 and within up‐to‐seven; B2 as a Child–Pugh score of 5–7 and out of up‐to‐seven; and B3 as a Child–Pugh score of 8–9, irrespective of tumor burden. The OS of patients in substage B1 who underwent LR was significantly better than that of patients receiving non‐surgical treatments, even after PSM. The OS of patients in substage B2 who underwent LR was significantly better than that of patients who underwent non‐surgical treatments; however, this difference disappeared after PSM. The OS of patients in substage B3 did not differ between treatment modalities. The authors concluded that LR provided better OS outcomes than non‐surgical treatments for substage B1 HCC patients, whereas OS was comparable between LR and non‐surgical treatments for substage B2 and B3 HCC patients [27].

In a Korean study enrolling 277 BCLC B HCC patients treated with either TACE (*n* = 225) or LR (*n* = 52), the patients were divided into four groups (B1–B4) based on tumor size and number, AFP level, and Child–Pugh score. LR resulted in better OS compared to TACE in the B2 group (*p* = 0.014) defined as having “oligo” (2–4) nodules and a tumor size of < 10 cm. OS did not differ significantly between LR and TACE in the other groups (B1 and B3) [28].

We believe that the up‐to‐seven criteria are not suitable for selecting patients with BCLC B HCC for LR because the advantage of resection is removal of large tumors; that is to say, tumor size does not matter when considering LR for patients with BCLC B HCC. In contrast, the impact of TACE on patients with large tumors is limited [2, 3]. For example, a patient with BCLC B HCC with two tumors, 6 and 2 cm in size, is classified as out of up‐to‐seven. We could perform LR for a large tumor, intraoperative RFA for a small tumor if the tumor is deeply located, or wedge resection if the tumor is superficially located. According to Spanish and Japanese studies [26, 27], such patients are not suitable for LR. In contrast, a patient with BCLC B HCC and multiple small tumors, up to 4 in number with the largest tumor 3 cm in size, located in one lobe of the liver is within up‐to‐seven. Although LR is technically feasible for removal of all tumors, TACE followed by RFA for residual tumors can also achieve a complete radiological response. In our experience, such a patient would not be recommended for LR despite being within up‐to‐seven and the ideal candidate for LR according to Spanish and Japanese studies [26, 27]. The results of the present study are similar to those of the Korean study, which recommended LR for patients with oligo‐tumors, that is, 2–4 tumors, whereas LR was not recommended for patients with multiple tumors, that is, ≥ 5 tumors [28].

In the present study, the median OS of both LR and TACE subgroups of the BCLC B2 group was around 2 years. According to the updated BCLC guidelines, the ideal BCLC stage B candidates for TACE are those with preserved liver function, good performance status, and well‐defined nodules. The expected OS is > 2.5 years [2]. Therefore, both LR and TACE may not be suitable for a proportion of BCLC B2 patients in the present study. Kudo et al. suggested that patients with BCLC B HCC and beyond up‐to‐seven criteria should undergo systemic therapies [29]. Similarly, Llovet et al. recommended systemic therapy for HCC patients with high tumor burden [30]. The updated BCLC guidelines recommend systemic therapies for BCLC B and diffuse infiltrative type HCC [2]. In summary, a proportion of patients with BCLC B2 in the present study may have diffuse infiltrative type HCC or high tumor burden HCC, which is not suitable for TACE [2, 29, 30]. However, because systemic therapies for BCLC B HCC were not reimbursed by Taiwan's National Health Insurance, very few patients with this tumor stage received such therapies.

The strength of the present study is its simplicity. By using two variables, image‐defined tumor number and the MELD score, we were able to preoperatively determine which subgroup of BCLC B HCC patients should undergo LR. However, the present study has some limitations. First, it is a retrospective and monocentric study. Second, the study lacked data on severe comorbidities. Although the OS of patients in the BCLC B1 subgroup undergoing LR was better than that of patients undergoing TACE, selection bias may exist as patients without severe comorbidities were selected for LR. Therefore, the results of the present study should be interpreted with caution. We did not perform PSM between LR and TACE because the two treatment modalities had similar characteristics except for the prevalence of anti‐HCV positivity, which was lower in patients undergoing LR than those receiving TACE (*p* = 0.046) in the BCLC B1 group. Whether the etiology of chronic liver disease is associated with the survival outcomes of BCLC B HCC patients undergoing LR is uncertain. Third, the study lacked data on cirrhosis, which is a well‐known variable associated with survival outcomes of patients with HCC undergoing LR [2]. In our HCC registry data, cirrhosis was defined based on the histology of patients undergoing surgery and on image studies of patients undergoing non‐surgical treatments. Image definition of cirrhosis can be vague and subjective; therefore, we do not present cirrhosis data. Fourth, this study lacked data on postoperative complications, information important for deciding the treatment modality.

In conclusion, we used two parameters, that is, ≤ 3 nodules and a MELD score of ≤ 9, to subclassify BCLC stage B HCC patients. BCLC B1 had to occur concomitantly with two parameters, whereas for BCLC B2, meeting only one criterion was sufficient. We found that patients with BCLC B1 are ideal candidates for LR, whereas those with BCLC B2 are not. The present study provides a simple and potentially clinically useful method of deciding the treatment modality for patients with BCLC B HCC and Child–Pugh Class A liver disease. External validation of our results is needed.

## Author Contributions

The conception or design of the work; or the acquisition, analysis, or interpretation of data for the work: Yi‐Hao Yen. Statistical analysis: Chih‐Yun Lin. Drafting the work: Yi‐Hao Yen. Reviewing it critically for important intellectual content: All authors. Final approval of the version to be published: All authors. Agreement to be accountable for all aspects of the work in ensuring that questions related to the accuracy or integrity of any part of the work are appropriately investigated and resolved: All authors.

## Funding

This study was supported by Chang Gung Memorial Hospital‐Kaohsiung Medical Center, Taiwan (Grants CORPG8N0271, CMRPG8L0181, and CMRPG8L0182).

## Ethics Statement

The Institutional Review Board of Kaohsiung Chang Gung Memorial Hospital approved this study (reference number: 202201189B0) and waived the need for informed consent.

## Conflicts of Interest

The authors declare no conflicts of interest.

## Data Availability

The raw data for the entire cohort are available via the following digital object identifier: https://www.dropbox.com/scl/fi/kcq4o2e38jtgw63ecjpzr/raw‐data‐LR‐vs‐TACE‐BCLC‐B.xlsx?rlkey=rk0yixbipscbsgt21zsdulf07&st=8q6n2o4j&dl=0.
